# Isolation and probiotic potential of lactic acid bacteria from swine feces for feed additive composition

**DOI:** 10.1007/s00203-021-02700-0

**Published:** 2021-12-23

**Authors:** Katarzyna Marchwińska, Daniela Gwiazdowska

**Affiliations:** Department of Natural Science and Quality Assurance, Institute of Quality Science, Poznań University of Economics and Business, Poznan, Poland

**Keywords:** Feed additives, Isolation, Lactic acid bacteria, Pigs, Probiotic

## Abstract

Animal microbiota is becoming an object of interest as a source of beneficial bacteria for commercial use. Moreover, the escalating problem of bacterial resistance to antibiotics is threatening animals and humans; therefore, in the last decade intensive search for alternative antimicrobials has been observed. In this study, lactic acid bacteria (LAB) were isolated from suckling and weaned pigs feces (376) and characterized to determine their functional properties and usability as pigs additives. Selection of the most promising LAB was made after each stage of research. Isolates were tested for their antimicrobial activity (376) and susceptibility to antibiotics (71). Selected LAB isolates (41) were tested for the production of organic acids, enzymatic activity, cell surface hydrophobicity and survival in gastrointestinal tract. Isolates selected for feed additive (5) were identified by MALDI-TOF mass spectrometry and partial sequence analysis of 16S rRNA gene, represented by *Lentilactobacillus, Lacticaseibacillus* (both previously classified as *Lactobacillus*) and *Pediococcus* genus. Feed additive prototype demonstrated high viability after lyophilization and during storage at 4 °C and − 20 °C for 30 days. Finally, feed additive was tested for survival in simulated alimentary tract of pigs, showing viability at the sufficient level to colonize the host. Studies are focused on obtaining beneficial strains of LAB with probiotic properties for pigs feed additive.

**Supplementary Information:**

The online version contains supplementary material available at 10.1007/s00203-021-02700-0.

## Introduction

The health-promoting effects of microorganisms have been the subject of intense research in the recent years, the possibilities of their usage are increasingly described (Braune and Blaut 2016; Bautista-Gallego et al. 2017; Hidalgo-Cantabrana et al. 2018). In animal husbandry, there is a great interest in using probiotic microorganisms as an alternative to antibiotics (Patterson and Burkholder 2003; Cheng et al. 2014). These antimicrobials used as animal growth promoters were banned in 2006 in European Union countries due to the prevalence of antibiotic-resistant microorganisms and possibility of transmission of pathogens to humans (European Parliament and the Council of the European Union 2003; Long et al. 2018). This particular injunction has prompted an increased interest in searching for new health and nutritional additives for animals (Dowarah et al. 2017).

The welfare of farmed animals is one of the elements taken into account in the framework of European Union (EU) policy, whose main goal is to provide consumers with safe food, ensuring that the food chain has a neutral or positive effect on the environment. Standards and legal regulations developed within its framework cover all stages of production, from breeding to consumption. Meanwhile, among the main threats in the food production chain, which pose a risk to human and animal health and life, are pathogenic microorganisms, their metabolites and, on the other hand overused antimicrobial substances (European Commission 2020). Management of animal livestock in an artificial environment requires the prevention of disease spreading as well as rapid weight gain. Therefore, probiotic bacteria have been increasingly used as supplements in the nutrition of farm animals of various species, mainly pigs, poultry, cows, sheep, horses as well as fur animals (Hill et al. 2014; Yang et al. 2015; Dowarah et al. 2018a).

Probiotics are defined by FAO/WHO as “live microorganisms that, when administered in adequate amounts, confer a health benefit on the host” (FAO/WHO 2002). The mechanism of impact of probiotic strains on animals is multidirectional and complex. Probiotic microorganisms change the dynamics of the microbial population in the digestive system by balancing the number of beneficial and harmful microbiota (Mountzouris et al. 2009; Thomas and Versalovic 2010). It is related with the production of antimicrobial substances, such as organic acids or bacteriocins (Shim et al. 2012). Organic acids, in particular lactic and acetic acids, inhibit the growth of pathogens, and reduce pH value of the environment to a lethal level for pathogenic bacteria (Commane et al. 2005; Fayol-Messaoudi et al. 2005; Daşkiran et al. 2012; He et al. 2019). Bacteriocins and other antimicrobial metabolites, such as hydrogen peroxide, also reduce the growth of undesirable microorganisms. In addition, probiotics show the ability to adhere to the intestinal epithelium and they can interfere with the communication processes of pathogenic bacteria, e.g. through the *quorum sensing* mechanism, what prevents their colonization (Medellin-Peña et al. 2007; Mookiah et al. 2014). The most common change in the qualitative composition of the intestinal microbial population consists in an increase of the number of LAB and a simultaneous decrease in the number of coliform bacteria, including *Escherichia coli* (Cao et al. 2013; Landy and Kavyani 2013; Mookiah et al. 2014; Zhang and Kim 2014; Spaiser et al. 2015). Literature data also indicate the possibility of non-specific stimulation of the immune system of animals, which is another beneficial effect of the use of probiotic microorganisms (Schrezenmeir and De Vrese 2001; Yousefi et al. 2019). Among the functional properties of probiotic bacteria, recent studies indicate the anti-cancer effect of these microorganisms. It is related to the competition within the microbiome of the digestive tract of animals and the limitation of the growth of bacteria producing fecal enzymes with a carcinogenic effect (Rautray et al. 2011; Siva Kumar et al. 2015). The composition of the bacterial population in the gastrointestinal tract (GIT), supported by the use of probiotics, is often associated with increased productivity of animals, affecting more efficient digestion and absorption of nutrients and increasing immunity. Disease control is also observed (Niba et al. 2009; Hung et al. 2012; Barba-Vidal et al. 2019; Xin et al. 2020).

LAB are considered as the most effective probiotics in swine production (Dowarah et al. 2018b; Sirichokchatchawan et al. 2018; He et al. 2019). However, for using LAB as feed additives some challenges must be met, one of which is that bacterial species must be generally recognised as safe (GRAS), as well as microorganisms must remain viable at the appropriate level during: processing, transport, storage conditions and passage through the digestive system of the animal. Moreover, many scientists underline that bacteria isolated from the host are more effective probiotics than isolates obtained from other sources (Chiang et al. 2015; Bautista-Gallego et al. 2017; Dowarah et al. 2017). Therefore, in the presented study, beneficial microorganisms were isolated from swine feces. Taking into account differentiated microbiota depending on the animal age, bacteria were isolated from feces of suckling and weaned pigs.

## Materials and methods

### Sample collection

The feces of ten suckling and ten weaned healthy pigs were collected by a veterinarian from a swine farm in Wielkopolska Province, Poland. The health status of the animals was evaluated by the veterinarian on the basis of general condition of piglet and no impairment of health especially lack occurrence of digestive disorders, which were monitored. The samples were stored in sterile conditions and transported to the laboratory in coolers with ice and processed immediately on the same day.

### Isolation of LAB

Piglets feces samples (10 g) were homogenized in stomacher BagMixer Interscience with 90 mL of sterile saline solution, serially diluted and cultured in MRS agar with the addition of 0.5% CaCO_3_ (Guo et al. 2010; Petsuriyawong and Khunajakr 2011), under microaerophilic conditions at 37 °C for 24 h (Torshizi et al. 2008; Adetoye et al. 2018). Randomly selected separated colonies were passaged onto MRS agar medium by reduction plating technique and incubated to obtain pure cultures. Individual colonies were propagated in MRS broth for further analysis. Isolated bacteria were stored as stocks in MRS broth at − 20 °C, with 80% glycerol in a 1:1 ratio (Adetoye et al. 2018). Every time before studies LAB were cultivated on MRS broth at 37 °C under microaerophilic conditions for 24 h.

### Antibacterial activity

Antibacterial susceptibility for 376 LAB isolates was tested using modified well diffusion method (Vu et al. 2002; Aujoulat et al. 2011). The indicator bacteria used for the studies were Gram-positive strains: *Clostridium perfringens* ATCC® 13124™, *Listeria monocytogenes* ATCC® 19115™, *Staphylococcus aureus* ATCC® 33862™ and Gram-negative strains: *Aeromonas hydrophila* ATCC® 7966™, *Campylobacter jejuni* ATCC® 33291™, *Enterobacter aerogenes* PCM 532, *Escherichia coli* ATCC® 8739™, *Proteus vulgaris* PCM 542, *Pseudomonas aeruginosa* ATCC® 9027™, *Salmonella enterica ser*. Enteritidis ATCC® 13076™, *Salmonella enterica ser*. Typhimurium PCM 2565, *Serratia marcescens* PCM 549, *Shigella flexneri* PCM 89 and *Yersinia enterocolitica* ATCC® 9610™. Tested indicator bacteria were obtained from American Type Culture Collection (ATCC) and Polish Collection of Microorganisms, Institute of Immunology and Experimental Therapy PAN (PCM). Suspensions in physiological saline with an optical density of 0.5 on the McFarland scale were prepared from 24 h cultures of indicator bacteria. Cultures were carried out using the flood technique, after solidifying the agar wells were cut out with sterile cork borer (diameter 10 mm). Next, freshly cultivated LAB cultures in the amount of 0.1 mL were introduced into wells (MRS broth as a control). The incubation conditions were in accordance with the optimum temperature for the growth of indicator bacteria: 30 °C or 37 °C for 24 h. *C. jejuni* and *C. perfringens* were incubated under microaerophilic and anaerobic conditions, respectively. The diameter of growth inhibition was measured, taking into account the well (10 mm) (Chen et al. 2014; Gwiazdowski et al. 2015; Islam et al. 2020). Based on the diameter of the inhibition zones, three activity ranges were established for LAB isolates with specific antibacterial properties: > 20.1 mm—strong activity; 20.0–15.1 mm—moderate activity and < 15.1 mm—weak activity.

Antibacterial activity was re-evaluated for 41 LAB isolates, selected on the basis of antibiotic susceptibility assessment, after 18 months of storage in refrigeration conditions at − 20 °C, with 80% glycerol in a 1:1 ratio. For the in vitro studies additional indicator microorganisms were tested: *A. hydrophila, S. flexneri, C. jejuni* and *P. aeruginosa.*

### Susceptibility to antibiotics

Antibiotic susceptibility of 71 LAB isolates was determined by the disk diffusion method using commercial antibiotic discs (Oxoid) (Torshizi et al. 2008; Pérez-Sánchez et al. 2011; Petsuriyawong and Khunajakr 2011). Ampicillin (10 mcg), vancomycin (30 mcg), gentamicin (10 mcg), kanamycin (30 mcg), streptomycin (300 mcg), erythromycin (15 mcg), clindamycin (2 mcg), tetracycline (30 mcg) and chloramphenicol (30 mcg) were used for the studies. As a criterion for the selection of antibiotics and their concentrations, data from the EFSA guidelines (European Food Safety Authority (EFSA) 2012) and thematic literature (Pérez-Sánchez et al. 2011; Ripamonti et al. 2011), as well as consultations with producers of feed additives for farm animals and breeders were used. Suspensions in saline solution were prepared from a 24 h LAB culture, setting the optical density at 1.0 on the McFarland scale. Samples were performed using the flood method, and after solidification of the medium, antibiotic discs were applied to the surface. Mueller–Hinton and MRS media were used in the study. Samples were incubated under anaerobic conditions with CO_2_ at 37 °C for 24 h. Controls were paper discs saturated with saline solution. The diameter of clear zones around the discs were measured, taking into account the size of the antibiotic discs (6 mm). The criteria for analysing the results of the sensitivity of microorganisms to the effects of antibiotics were determined using The Clinical and Laboratory Standards Institute (CLSI) M100 document (29th edition) (Clinical and Laboratory Standards Institute (CLSI) 2019), Charteris et al. (Charteris et al. 1998, 1999) and Han et al. (2015) dividing LAB to the following categories: resistant (R), intermediate susceptibility (I) and sensitive (S).

Susceptibility to antibiotics using the broth microdilution assay following the standard procedure of the ISO 10932|IDF 223:2010 (International Organization for Standardization (ISO) 2010) recommended by the EFSA (European Food Safety Authority (EFSA) 2012), was re-evaluated for five LAB isolates, selected on the basis of antibacterial activity, antibiotic susceptibility and functional properties. Minimum inhibitory concentration (MIC) of the following antibiotics: ampicillin (Sigma–Aldrich, Germany), gentamicin (Sigma–Aldrich, Germany), kanamycin (BioShop, Canada), streptomycin (Fisher Scientific, Belgium), erythromycin (ThermoFisher, Germany), clindamycin (ThermoFisher, Germany), tetracycline (Fisher Scientific, Belgium) and chloramphenicol (BioShop, Canada), was determined (European Food Safety Authority (EFSA) 2012). According to the guidance, determination of vancomycin microbiological cut-off values is not required for obligate heterofermentative *Lactobacillus* sp. (e.g. *L. buchneri*), *L. paracasei* and *Pediococcus* sp., therefore, it was not included in the research (European Food Safety Authority (EFSA) 2012). Each tested antibiotic was diluted into LSM medium under the appropriate concentration. An aliquot of 100 μL of twofold dilutions of tested antimicrobials in the concentration ranging from 0.008 to 128 μg/mL, depending on the antibiotic, were prepared in U-bottom 96-well microtiter plates. Each antibiotic was diluted into LAB susceptibility test medium (LSM) composed of Iso-Sensitest (Oxoid, Canada) broth and MRS broth (ratio 9:1, respectively) under appropriate concentration. The bacterial suspensions in the LSM from 24 h cultures were standardized to obtain density 1 McFarland’s standard which refers to the spectrophotometric equivalent of 3 × 10^8^ CFU/mL, further diluted 1000-fold (Muñoz-Atienza et al. 2013) Next, the microplates were inoculated with the 100 μL of bacterial suspension and incubated at a temperature 37 °C for 24 h. The negative control was the sterile LSM medium with the addition of antibiotics, the positive control was the standardized bacterial inoculum without the inhibitory agent addition. Control strain used for the tests was *E. coli* ATCC 25922. After 24 h of incubation, the microorganisms' growth optical density was determined at 600 nm using BioTek Epoch 2 microplate reader. The MIC value was defined as the lowest antibiotic concentration, inhibiting the bacterial growth. The tested LAB results were further on interpreted based on the EFSA guidance (European Food Safety Authority (EFSA) 2012), distinguishing between sustainable and resistant strains.

### Functional properties of LAB isolates

#### Production of selected organic acids

Determination of lactic, acetic, succinic and propionic acids content in 24 h bacterial cultures of 41 isolates on MRS broth was performed by high-performance liquid chromatography (HPLC) (Lim and Lee 2013; Gwiazdowski et al. 2015). The amount of 2 mL culture samples were centrifuged in sterile Eppendorf centrifuge tubes (10,000 rpm/min, 10 min) in a Centrifuge 5804R, Eppendorf AG. The supernatants were filtered through Millex®-LCR 0.22 Pm filters (Millipore). The tests were performed on a 2695 Waters liquid chromatograph coupled with a 2414 Refractive Index (RI) detector (Waters, Milford, MA, USA). Aminex HPX-87H 300 × 7.8 mm columns (BIO-RAD) were used for the determinations. The eluent was 0.004 M H_2_SO_4_ solution, with a flow of 0.75 mL/min. Solutions of lactic, acetic, succinic and propionic acids with a concentration of 0.1 µg/µL were used as standards. The determination was carried out at a temperature of 65 °C. Identification was made using an external standard method with the use of peak areas.

#### Enzymatic activity assay

The amount of 41 LAB isolates selected for further studies were tested for their amylase, protease and lipase activity based on the modified methods of Taheri et al. (2009), Guo et al.(2010) and Moslehishad et al. (2013). LAB were spot-inoculated on: modified MRS agar with 2% of starch for amylolytic activity, agar with 1% of skimmed milk for proteolytic activity and agar with Tween 80 and calcium chloride for lipolytic activity. Enzymatic properties were assessed on the basis of halo zones (amylolytic) and turbidity zones (lipolytic and proteolytic) around the colony growth after incubation under anaerobic conditions with CO_2_ at 37 °C for 24 h. To detect clear zones of amylase activity, Lugol’s solution was poured over the plates.

#### Cell surface hydrophobicity test

The cell surface hydrophobicity was determined for 41 LAB according to Taheri et al. (2009) and modified Pérez-Sánchez et al. (2011). LAB isolates were centrifuged for 5 min at 7500 rpm/min in a Centrifuge 5804R, Eppendorf AG. The supernatant was removed, the bacterial cells were washed twice with phosphate buffer and resuspended in physiological saline to obtain an optical density of 0.5 at 600 nm (OD_600_). The bacterial cell suspension in the amount of 3 mL was transferred into sterile tubes and 1 mL of toluene was added. Samples were shaken using a Vortex shaker for 90 s. The tubes were then allowed to stand for 15 min to separate the phases. Finally, the absorbance of the aqueous phase was measured at 600 nm. The hydrophobicity of the cells was calculated as the average percentage decrease in density of the LAB suspension (OD_600_) due to the adhesion of microorganisms to the hydrocarbon used.

#### Bile salts tolerance

The effect of bile salts on the survival of the 41 LAB isolates was tested according to a modified methods of Lin et al. (2007) and Guo et al. (2010) using microtiter plates. Twofold dilutions of 2% ox bile solution were prepared using MRS broth as diluent, followed by the addition of 100 µl bacterial suspension. The final concentration of ox bile in the cultures was 1.00%, 0.50% and 0.25%. Incubation was carried out under anaerobic conditions obtained using CO_2_ at 37 °C for 24 h. Spectrophotometric measurements were made using the BioTek Instruments EPOCH 2 microplate reader. Absorbance was measured at 600 nm (OD_600_) immediately after the samples were prepared and at 1, 3 and 24 h of incubation. The negative control was medium without LAB inoculum. A positive control was bacterial culture in MRS medium without the addition of ox bile. Strains that survived the bile salts exposure in > 50% after 24 h were considered as well tolerating the GIT conditions. In the range from 80 to 50% bile salts tolerance the LAB isolates were considered as well tolerating and above 80% as very well tolerating the GIT component.

#### Acidic pH tolerance

The impact of the environment pH on the survival of the 41 LAB isolates was tested with modified Guo et al. assay (Guo et al. 2010) using microtiter plates. Bacteria were incubated in the MRS medium with pH set at the levels 2 and 3, using hydrochloric acid (Pérez-Sánchez et al. 2011). Incubation was carried out under anaerobic conditions with CO_2_ at 37 °C for 24 h. Spectrophotometric measurements were made using the BioTek Instruments EPOCH 2 microplate reader. Absorbance was measured at 600 nm (OD_600_) immediately after the samples were prepared and after 1, 3 and 24 h of incubation. The negative control was medium without inoculum. The positive control was bacterial culture in MRS medium pH 6.5. Three tolerance ranges were established for LAB survival in 2.0 and 3.0 pH values after 24 h of incubation depending of percentage: > 50% viability—well tolerating isolates; 50–30% viability—moderate tolerating isolates and < 30% viability—weak tolerating isolates.

### LAB identification

#### MALDI-TOF mass spectrometry

Selected, on the basis of obtained results, five LAB isolates were determined using MALDI-TOF Microflex mass spectrometry (Bruker, Germany) according to the standard producers protocol (Dec et al. 2014, 2016). The obtained LAB spectra have been identified using the data stored in BioTyper reference library of MALDI-TOF mass spectra and NCBI (The National Center for Biotechnology Information). Bruker MALDI-TOF BioTyper criteria for interpretation of the results: for the high-confidence of results, the value of the identification index must score ≥ 2. The range from 1.99 to 1.70 indicates low-confidence identification, the value lower than 1.70 equals lack of microorganism identification.

#### Genetic identification

Identification of five selected LAB isolates was carried out based on the partial sequence analysis of the 16S rRNA gene, which required the isolation of genomic DNA, PCR with appropriately selected primers, sequencing and the analysis of the obtained sequences. 16S rRNA genes of the selected LAB strains were amplified using primers 1492r (ggT TAC CTT gTT ACg ACT T) and S-D-Bact-0008 (AgA gTT TgA TCM Tgg CTC AG) (Leser et al. 2002; Pang et al. 2011). The 1500 bases sequences were edited, combined and generated using the GeneDoc 2.700. Obtained sequences were analyzed using the BLAST (Megablast algorithm) Tool (https://blast.ncbi.nlm.nih.gov/) and submitted in GenBank. The unrooted phylogenetic tree was constructed to determine the closest LAB species by the neighbor-joining method (Saitou and Imanishi 1989) using the MEGA X software (Kumar et al. 2018). For the construction of the phylogenetic tree, 16S rRNA sequences of *L. paracasei* (GeneBank ID: MZ411515.1, M411523.1, MZ411532.1, NR_025880.1), *L. paracasei subsp. paracasei* (GeneBank ID: LC096209.1), *L. paracasei subsp. tolerans* (GeneBank ID: LC065035.1), *P. pentosaceus* (GeneBank ID: MW025983.1, KX886792.1), *L. buchneri* (GeneBank ID: MW025972.1, NR_041293.1) and *E. coli* (GeneBank ID: X80725) were obtained from NCBI database.

### Feed additive preparation

#### Preparation of freeze-dried bacterial cultures

Lyophilization of selected bacterial isolates cultured on a skim milk carrier was carried out on a medium enriched with maltodextrin and trehalose (Meng et al. 2008; Strasser et al. 2009; Lo Verso et al. 2018). Experimental work was performed using a shelf freeze dryer, Alpha 1 D series. Samples of 24 h cultures of each isolate separately, in the amount of 20 mL, were transferred to sterile disposable containers of polypropylene with a capacity of 60 mL. The test samples were then kept in the freezer at − 20 °C for 72 h, followed by lyophilization. The process of vacuum freeze drying took place at constant parameters: pressure 63 Pa, time 96 h, temperature of the heating shelves of the freeze dryer 30 °C.

#### Assessment of the stability of freeze-dried bacterial cultures and additive during storage

Samples of five lyophilized bacterial cultures were grounded and mixed thoroughly in equal proportions, giving the prototype of feed additive. The stability of five freeze-dried bacterial cultures and the feed additive was determined by a plating method comparing the number of bacteria immediately after freeze drying and after 30 days of storage at 4 °C and − 22 °C. Samples were kept in sealed, sterile containers to assess storage stability (Fonseca et al. 2006, 2015a; Strasser et al. 2009).

### Evaluation of the survival of LAB present in feed additive in the simulated digestive system

The survival of LAB strains in a simulated digestive system was determined using in vitro pig model. The survival of bacteria was evaluated by transferring test samples: (A) lyophilized mix of five LAB cultures contained addiction of feed and (B) lyophilized mix of five LAB cultures, through the simulated GIT. In the experiments, commercial feed was used—a universal protein concentrate for pigs during the fattening period. This mix is made on the basis of, among others, cereals, high-protein extraction shots, vitamin and mineral premix, mineral additives as well as biologically active compounds and other components. Sample A was 5 g of protein concentrate with 10% of the freeze-dried LAB, sample B was 5 g of the freeze-dried LAB. The in vitro digestion model included a three-stage digestion process: in the mouth (simulated saliva solution: sodium chloride, potassium chloride, calcium chloride, sodium bicarbonate, α-amylase, water) (Avantaggiato et al. 2003; Versantvoort et al. 2005), stomach (0.1 M phosphate buffer; 0.2 M hydrochloric acid solution, water pepsin 25 mg/cm^3^ solution) and small intestine (water pancreatin 100 mg/cm^3^ solution, 0.6 M sodium hydroxide solution, 0.2 M phosphate buffer) (Boisen and Fernández 1997). Samples A and B were pre-digested at 39 °C in simulated solution of saliva for 5 min, then in simulated gastric juice for 2 h and intestinal juice for 4 h. At each stage, the test mixture was incubated at 39 °C under continuous mixing at 150 rpm on a rotary shaker to ensure homogeneous distribution of all components (Boisen and Fernández 1997; Avantaggiato et al. 2003; Versantvoort et al. 2005). At the beginning of each section of the GIT and after the retention time, the viability of LAB was determined.

### Statistical analysis

Statistical analysis of the results was carried out using SPSS Statistics 23 and Microsoft Excel®. All the experiments were carried out in three parallel replicates. The standard deviation value was calculated for the obtained results. One-way analysis of variance (ANOVA) was performed to compare the mean values. The significance of differences between the mean values of the results was determined by the Tukey post hoc test. All statistical hypotheses were verified at the significance level *p* < 0.05.

## Results

### Isolation of LAB

A total of 244 microorganisms were isolated from the feces of suckling piglets and 132 from weaned piglets. All isolates were preliminary identified as LAB based on the criteria of being Gram-positive and catalase negative rods and cocci, hydrolysing CaCO_3_ on the MRS agar plates. The number of LAB in tested feces samples ranged from 7.08 to 9.85 log CFU/g from suckling piglets and from 8.46 up to 9.99 log CFU/g from weaned piglets.

### Antibacterial activity

Antibacterial activity was assessed towards chosen pathogens of the intestinal tract of pigs, human pathogens and some potentially pathogenic microorganisms. The results of in vitro experiments showed a large diversity of the 376 LAB isolates antibacterial effect, both taking into account their activity spectrum and the degree of inhibition growth of indicator microorganisms (Table S1 in Supplementary material). Out of all the tested bacterial isolates, 87 did not exhibit any antagonistic properties against the indicator microorganisms. Most isolates that showed antibacterial activity were characterized by a weak interaction (8.24–21.01% depending on the indicator strain). Only a small group of isolates was characterized by moderate (0.27–11.17%) and strong (0.00–4.52%) activity towards indicator microorganisms. Based on the results of antibacterial activity, 71 LAB isolates with the strongest antimicrobial effect towards different indicator bacteria were selected for further tests.

**Fig. 1:**
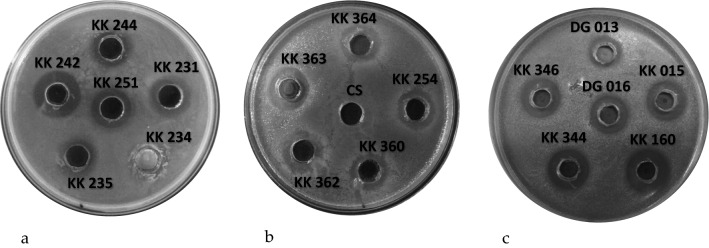
Antibacterial activity of LAB isolates against: **a** *Salmonella enterica* ser. Typhimurium; **b** *Serratia marcescens*; **c** *Staphylococcus aureus.* CS, control sample (MRS broth); KK 231-DG 016, numbers of tested LAB isolates

After 18 months of storage, 41 LAB isolates, selected on the basis of susceptibility to antibiotics, were redefined for maintaining their antibacterial activity towards an extended group of indicator bacteria (Fig. 1). LAB isolates showed a similar antagonistic effect against indicator bacteria, compared to the results obtained in previous antimicrobial tests. Only in the case of antagonistic effect towards *C. perfringens*, after a storage period, a significant reduction in the activity of most LAB isolates was observed. Out of the 41 tested isolates, only 2 of them inhibited the growth of *C. perfringens* to a small extent. The results of the experiments also showed a diverse antimicrobial effect of the tested LAB, taking into account both their activity spectrum and the degree of growth inhibition. Furthermore, 4 indicator microorganisms were added for antibacterial evaluation. *A. hydrophila* and *P. aeruginosa* were susceptible to 40 out of 41 tested isolates, with growth inhibition ranging from low to high. All tested isolates showed antagonistic activity against *S. flexneri,* where the values ​​of growth inhibition zones ranged from low impact to moderate. Inhibition of *C. jejuni* growth was also observed by 30 tested LAB, where high antimicrobial activity was observed in several isolates. In summary, the conducted research indicates the ability of LAB to inhibit the growth of *A. hydrophila, S. flexneri, C. jejuni* and *P. aeruginosa.* In most cases, the antibacterial activity of the tested isolates was maintained after 18 months, although, as mentioned, a reduction, disappearance and even increase in the activity was observed in relation to selected indicator microorganisms. Detailed results concerning antibacterial activity are presented for five finally chosen LAB isolates in Table 3.

### Susceptibility to antibiotics

The assessment of susceptibility to nine antibiotics was the next step of LAB selection. Using the antibiogram method to determine the drug resistance of LAB, the following levels of action were specified: sensitive isolate, intermediate sensitive isolate or resistant isolate, depending on the size of the growth inhibition zones. The sensitivity of selected 71 LAB isolates to chosen antibiotics was varied (Table S2 in Supplementary material). All the tested isolates were vulnerable to the effects of clindamycin and erythromycin (in the tested concentrations), except for one isolate derived from the feces of suckling piglets, which showed medium sensitivity to erythromycin. Most isolates were also sensitive to chloramphenicol as only one isolate showed moderate sensitivity. Among LAB, five showed intermediate susceptibility towards tetracycline and one resistance. 52 isolates showed high sensitivity to 10 mcg ampicillin, while 18 isolates—medium sensitivity, as well as lack of sensitivity was observed for 1 isolate. The number of LAB isolates showing high or medium sensitivity to streptomycin at 300 mcg was 67. In case of gentamicin, a wide variation of isolates sensitivity to this antibiotic was observed. 50 LAB isolates were sensitive, including 12 vulnerability, while 20 isolates were insensitive to gentamicin at the tested concentration. The highest number of resistant isolates was found for kanamycin (33) and vancomycin (69) at 30 mcg. Based on the obtained results, 41 LAB isolates, which had no or low acceptable antibiotic resistance, were selected for further studies. Among the bacteria selected after this stage, 29 isolates came from the digestive tract of suckling piglets and 12 isolates from the weaned piglets.

### Production of selected organic acids

The selected 41 LAB were characterized by a diverse content of lactic acid in culture, which was the dominant product and ranged from 7.67 to 40.68 mg/mL. Among LAB isolates, 17 had a high level of lactic acid production, in the range from 26.31 to 40.68 mg/mL. In cultures of 22 isolates, the amount of lactic acid was at an average level, from 15.04 to 24.62 mg/mL, and in 2 cases the content of this metabolite was low and ranged from 7.67 to 12.76 mg/mL. A small amount of acetic acid was found in the LAB cultures, the concentration was between 2.59 and 4.79 mg/mL. It should be emphasized that the acetic acid content varied significantly in most cases. Tested LAB isolates did not produce propionic and succinic acids.

### Functional properties of LAB

#### Enzymatic activities

Enzymatic activity tests included proteolytic, amylolytic and lipolytic activity (Table 1). The results of the studies showed that all tested LAB isolates caused the degradation of milk casein (clear zones around the growth). Among tested LAB, 46% showed starch degradation (clear zone after flooding with Lugol's liquid). Amylolytic properties were noted for 13 isolates derived from the suckling piglets feces and 6 isolates isolated from the weaned piglets feces. When assessing the ability of the tested LAB to produce lipases, no halo zones were found around the bacterial colonies, which means that LAB isolates did not exhibit lipolytic properties.

**Table 1:** Chosen properties of 41 selected LAB strains

Enzymatic properties^n^				Hydrophobicity^n^		GIT conditions^n,2^						
						Bile salts				pH value		
	Proteolytic	Amylolytic	Lipolytic				0.25%	0.50%	1.00%		2	3
Zones value				Degree of adhesion								
3–2 mm	5	0	0	Good (> 40%)	7	High tolerance (> 80%)^3^	20	9	17	Good tolerance (> 50%)^3^	12	14
1 mm	36	19	0	Moderate (40–20%)	12	Good tolerance (80–50%)^3^	17	25	20	Moderate tolerance (50–30%)^3^	25	26
ND^1^	0	22	41	Weak (< 20%)	22	Moderate tolerance (< 50%)^3^	4	7	4	Weak tolerance (< 30%)^3^	4	1

^n^Number of LAB isolates

^1^ND—enzymatic activity not detected

^2^Survival of 41 selected LAB isolates in the GIT conditions after 24 h of incubation

^3^Percentage of LAB viability in relation to control sample

#### Cell surface hydrophobicity test

The ability of LAB cells to adhere to hydrocarbons as an indicator of their hydrophobicity, and also the criterion of the ability of adhesion to intestinal epithelial cells was tested. The results are expressed in the form of percentage changes in the density of the bacterial cell suspension after adhesion to the hydrocarbon used. Above 40% of the value of toluene adhesion was assumed as a high degree of cell hydrophobicity, ranging from 20% to 40%, as medium, while low level, below 20%, was determined (Table 1). The results regarding the assessment of the hydrophobicity of LAB derived from piglets feces indicate a large variation in this parameter. Out of the 41 tested isolates, 7 were characterized by high and 11—by medium hydrophobicity values from 22% to 63%. The remaining 22 isolates had a low toluene adhesion value, no more than 10%.

#### Survival of LAB in the GIT conditions: bile salts and acidic pH tolerance

The presence of bile salts in the samples had a different effect on the growth of the tested isolates (Table 1). Detailed data concerning selected strains are presented in Table 5 and on Fig. 3. Tolerance ranges presented in the Table 1 are established by the Authors based on the thematic literature data (García-Hernández et al. 2016) as well as obtained results. Bile salts tolerance, expressed as a percentage of population growth in the presence of this component relative to the control sample, depended on the tested LAB isolate as well as the concentration of ox bile salts in the culture. As a rule, the increase in bile salt content caused a decrease in bacterial survival rate; however, for many isolates, there were no significant differences in growth in the presence of 0.5 and 1%. Several isolates (e.g. KK 033) showed very high tolerance to the presence of bile salts, resulting in intense growth comparable to bacterial growth in control samples. Significant growth inhibition of some LAB (e.g. DG 068) was also noted. Similar relationships were observed in the group of isolates derived from the suckling and weaned piglets feces. For some isolates, the effect of bile salts concentration on population growth was visible in the first hours. The growth of most isolates remained at a similar level during the first hours of incubation, regardless of concentration, while differences in growth were only revealed in the following hours. Isolates that showed low tolerance of this component in the medium were characterized by weak growth during the first hours.

Taking into account the pH range prevailing in the stomach of various monogastric animals, the survival of the tested LAB isolates was tested in MRS medium, whose pH was set at 2.0 and 3.0. For comparison, cultures were run in parallel under optimal conditions at a pH of 6.5. It was found that the OD_600_ value after 24 h incubation for all isolates was significantly lower in the medium at pH 2.0 and 3.0 compared to the control value. However, a diverse tolerance of the tested LAB to low pH values was found (Table 1). Statistical analysis showed significant differences between isolates. Comparing bacterial growth at pH 2 and pH 3, in a few cases significant differences in OD_600_ values can be seen, which illustrates the growth of isolates under these conditions. The weaker growth was also observed at pH 2 compared to pH 3.

### Identification of selected LAB isolates

The conducted research including production of organic acids, enzymatic activity, cell surface hydrophobicity as well as bile salts and acidic pH tolerance, allowed the selection of five LAB isolates demonstrating high antimicrobial activity, sensitivity to antibiotics, high production values of lactic acid as well as good survival in GIT conditions. Moreover, the obtained results of five selected LAB isolates were meant to complement each other in terms of the examined features. Selected LAB isolates were identified by MALDI-TOF mass spectrometry and sequencing of the 16S rRNA gene. The partial sequencing results obtained using two complementary primers 1492r and S-D-Bact-0008, after initial editing in GeneDoc 2.7.000, were combined. Sequence similarity searching was carried out using the BLAST algorithm. Results covering LAB genus and identification value are compiled in Table 2. In the group of LAB derived from the suckling piglets feces, isolates belonged to the following species *Lacticaseibacillus* *paracasei, Lentilactobacillus buchneri* and *Pediococcus pentosaceus*. One selected isolate obtained from weaned piglets showed homology to *L. paracasei*. The data restrictions of GeneBank were observed for three strains which have been identified as *L. paracasei* species, with differences on subspecies level identification. Query coverage of the presented LAB identification results obtained using BLAST was 99–100%. The combined partial sequences of isolates have been deposited in the GenBank.

**Table 2:** Identification of five selected LAB isolates by MALDI-TOF mass spectrometry and 16S rRNA gene sequence

LAB no	MALDI-TOF Identification		16S rRNA gene sequencing		Results
			BLAST		
	Reference strain (NCBI strain no.)	LS value^1,2^	Reference strain (GeneBank ID)	ID value^3^ (%)	
Isolation source: Suckling piglets					
KK 008	*L. paracasei* (47714)	2.39	*L. paracasei* (NR_025880.1)	99.72	*L. paracasei* (GeneBank ID: MZ411515.1)
			*L. paracasei subsp. paracasei* (LC096209.1)	99.72	
			*L. paracasei subsp. tolerans* (LC065035.1)	99.65	
KK 033	*L. paracasei* (47714)	2.14	*L. paracasei* (NR_025880.1)	99.79	*L. paracasei* (GeneBank ID: MZ411523.1)
			*L. paracasei subsp. paracasei* (LC096209.1)	99.72	
DG 059	*P. pentosaceus* (1255)	2.39	*P. pentosaceus* (KX886792.1)	99.93	*P. pentosaceus* (GeneBank ID: MW025983.1)
DG 068	*L. buchneri* (1581)	2.02	*L. buchneri* (NR_041293.1)	99.86%	*L. buchneri* (GeneBank ID: MW025972.1)
Isolation source: Weaned piglet					
KK 160	*L. paracasei* (47714)	2.49	*L. paracasei* (NR_025880.1)	99.86	*L. paracasei* (GeneBank ID: MZ411532.1)
			*L. paracasei subsp. paracasei* (LC096209.1)	99.79	

^1^LS value—BioTyper Log score value

^2^The significance of the identification index according to Bruker MALDI Biotyper: range ≥ 2.00—high confidence identification

^3^ID value—identification value

**Fig. 2:**
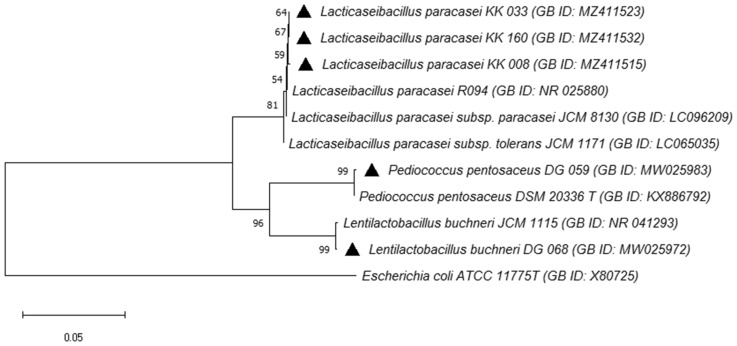
Phylogenetic tree based on 16S rRNA gene sequences showing the position of five finally selected LAB isolates. *E. coli* ATCC 11775T was taken as an out-group. Bootstrap values are given at branching points. Filled upward triangle, selected LAB strains isolated in this paper

For LAB strains identification and specific species confirmation, molecular phylogeny analysis was performed, the phylogenetic tree was constructed based on 16S rRNA sequences using by neighbor-joining method (no. of bootstrap replications = 2000) (Fig. 2) (Felsenstein 1985; Saitou and Imanishi 1989; Tamura et al. 2004; Kumar et al. 2018). Following the phylogenetic analysis, LAB strains KK 008, KK 033, and KK 160 were placed in the cluster making up the *Lacticaseibacillus* genus, subgroup *L. paracasei*. The strain DG 068 was placed in the *Lentilactobacillus* cluster, *L. buchneri* subgroup. The strain DG 059 was positioned in the *Pediococcus* genus cluster, subgroup *P. pentosaceus*. The conducted phylogenetic analysis also confirmed that the results of identification studies for the isolated LAB strains were correct.

### Characteristics of five finally selected LAB strains

**Fig. 3:**
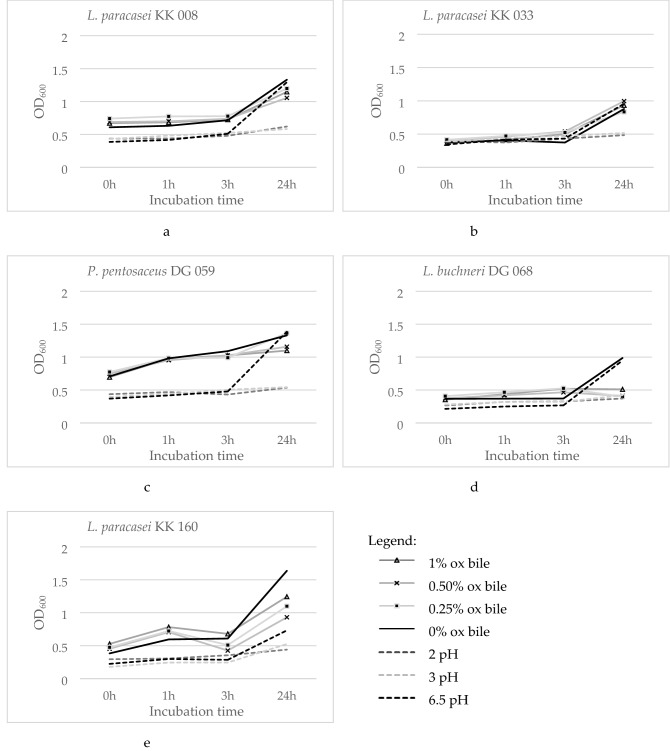
LAB survival in the GIT: bile salts and acidic pH tolerance: **a** *L. paracasei* KK 008; **b** *L. paracasei* KK 033; **c** *P. pentosaceus* DG 059; **d** *L. buchneri* DG 068; **e** *L. paracasei* KK 160

The results of laboratory experiments carried out to the point of feed additive composition, obtained for five selected bacterial strains, are summarized in Tables 3, 4, 5, as well as on Fig. 3. The feed additive developed for pigs supplementation included the following bacteria isolates: *L. paracasei* KK 008, *L. paracasei* KK 033, *L. paracasei* KK 160, *P. pentosaceus* DG 059 and *L. buchneri* DG 068. Selected isolates showed a wide range of antibacterial activity, which was tested twice: first, as the bacteria were isolated and identified as LAB and second after 18 months of storage. Differences in demonstrated antagonistic activity between the months have been observed. Both weaker antibacterial activity or its loss as well as gaining and greater antagonistic properties were spotted. None of five selected LAB isolates inhibited the growth of *Y. enterocolitica*. Furthermore, LAB isolates after 18 months of storage were tested positive for the inhibition of *A. hydrophila*, *C. jejuni*, *P. aeruginosa* and *S. fexneri.*

**Table 3:** Antibacterial activity of selected LAB strains

Strains	Storage (months)	The diameter of the growth inhibition zone (mm)^1^				
		KK 008	KK 033	DG 059	DG 068	KK 160
*C. perfringens*	0	17.33 ± 0.94	16.00 ± 0.00	16.67 ± 0.94	12.67 ± 0.94	20.00 ± 0.00
	18	ND	ND	ND	11.00 ± 0.00	ND
*L. monocytogenes*	0	14.67 ± 0.94	14.00 ± 0.00	15.00 ± 0.00	15.00 ± 0.00	12.00 ± 0.00
	18	13.33 ± 0.47	14.00 ± 0.00	14.33 ± 0.47	15.33 ± 0.47	16.00 ± 0.00
*S. aureus*	0	20.00 ± 0.00	18.00 ± 0.00	14.00 ± 0.00	14.00 ± 0.00	24.00 ± 0.00
	18	15.33 ± 0.47	15.33 ± 0.94	ND	16.33 ± 0.47	15.00 ± 0.00
*E. aerogenes*	0	18.00 ± 0.00	16.00 ± 0.00	16.00 ± 0.00	16.00 ± 0.00	ND
	18	13.67 ± 0.94	11.00 ± 0.00	ND	11.33 ± 0.47	16.67 ± 0.47
*E. coli*	0	18.00 ± 0.00	16.00 ± 0.00	16.00 ± 0.00	16.50 ± 0.00	15.67 ± 1.89
	18	18.33 ± 0.94	17.00 ± 0.00	14.67 ± 0.47	16.67 ± 0.47	18.00 ± 0.00
*P. vulgaris*	0	13.33 ± 0.94	12.67 ± 0.94	ND	ND	12.67 ± 0.94
	18	16.67 ± 0.47	15.00 ± 0.00	13.33 ± 0.47	12.33 ± 0.47	15.33 ± 0.47
*S. enterica* ser. Enteritidis	0	12.00 ± 0.00	11.33 ± 0.94	ND	17.00 ± 0.00	14.33 ± 0.47
	18	13.33 ± 0.47	12.67 ± 0.94	ND	18.67 ± 0.47	13.67 ± 0.94
*S. enterica* ser. Typhimurium	0	16.67 ± 1.89	16.67 ± 0.94	18.00 ± 0.94	14.67 ± 0.94	12.00 ± 0.00
	18	18.00 ± 0.00	16.67 ± 0.47	ND	16.33 ± 0.47	13.67 ± 0.94
*S. marcescens*	0	22.67 ± 4.71	18.00 ± 0.00	14.67 ± 0.94	12.67 ± 0.94	15.00 ± 1.41
	18	15.67 ± 0.47	15.00 ± 0.00	12.00 ± 0.00	15.67 ± 0.47	14.67 ± 0.47
*Y. enterocolitica*	0	ND	ND	ND	ND	ND
	18	ND	ND	ND	ND	ND
*A. hydrophila*	18	20.67 ± 0.47	16.33 ± 0.47	16.33 ± 1.89	17.00 ± 0.00	21.00 ± 1.41
*C. jejuni*	18	ND	11.33 ± 0.94	13.00 ± 1.41	11.00 ± 0.00	15.33 ± 0.47
*P. aeruginosa*	18	15.00 ± 1.41	16.67 ± 0.94	13.67 ± 0.94	13.67 ± 0.47	16.33 ± 0.47
*S. flexneri*	18	13.67 ± 0.47	13.00 ± 0.00	11.00 ± 0.00	18.33 ± 0.47	13.67 ± 0.94

^1^Including the diameter of 10 mm well; KK 008—*L. paracasei*; KK 033—*L. paracasei*; DG 059—*P. pentosaceus*; DG 068—*L. buchneri*; KK 160—*L. paracasei*; ND—inhibition zones not detected

**Table 4:** MIC distribution of five selected LAB strains

Antibiotics	AMP	CN	K	S	E	DA	TE	C
Dilution ranges (µg/mL)	0.016–32	0.03–64	0.06–128	0.06–128	0.008–16	0.008–16	0.016–32	0.008–16
Isolate no	MIC values (µg/mL)^1^							
*L. paracasei* KK 008	1 (4)	8 (32)	8 (64)	4 (64)	< 0.008 (1)	< 0.008 (1)	0.03 (4)	1 (4)
*L. paracasei* KK 033	1 (4)	8 (32)	8 (64)	4 (64)	< 0.008 (1)	< 0.008 (1)	0.03 (4)	1 (4)
*P. pentosaceus* DG 059	1 (4)	8 (16)	8 (64)	4 (64)	< 0.008 (1)	< 0.008 (1)	0.016 (8)	0.5 (4)
*L. buchneri* DG 068	0.5 (2)	8 (16)	8 (32)	4 (64)	< 0.008 (1)	< 0.008 (1)	0.016 (8)	0.5 (4)
*L. paracasei* KK 160	1 (4)	16 (32)	8 (64)	4 (64)	< 0.008 (1)	< 0.008 (1)	0.03 (4)	1 (4)

^1^EFSA breakpoints (mg/L) for each LAB isolate are presented in the brackets, isolates with the MIC higher than the EFSA breakpoint value are considered as resistant strains. Inhibitors of cell wall synthesis: AMP, ampicillin; VA, vancomycin; Inhibitors of protein synthesis: CN, gentamycin; K, kanamycin; S, streptomycin; E, erythromycin; DA, clindamycin; T, tetracycline; C, chloramphenicol

**Table 5:** Functional properties of five selected LAB strains

Isolate no	Lactic acid	Hydrophobicity	Acid tolerance		Bile salts tolerance		
			pH 2.0	pH 3.0	0.25% oxgall	0.5% oxgall	1.0% oxgall
	(mg/mL)	(%)	Percentage of viability after 24 h incubation (%)				
KK 008	31.69 ± 0.07	29.01 ± 1.10	47.95 ± 0.48	45.01 ± 1.37	90.01 ± 0.83	79.26 ± 0.38	86.18 ± 1.66
KK 033	19.47 ± 0.07	23.18 ± 0,50	50.63 ± 0.41	53.97 ± 1.16	95.10 ± 0.60	113.44 ± 0.40	106.26 ± 1.39
DG 059	15.15 ± 0.06	22.44 ± 1.50	39.20 ± 0.54	44.36 ± 0.95	40.53 ± 0.25	41.74 ± 0.24	51.77 ± 0.78
DG 068	16.67 ± 0.07	41.61 ± 0.87	60.11 ± 0.23	71.58 ± 1.34	67.20 ± 0.73	56.87 ± 1.32	75.99 ± 1.85
KK 160	24.62 ± 0.06	23.23 ± 2.06	30.74 ± 0.37	39.32 ± 1.28	74.06 ± 0.71	77.14 ± 0.39	102.63 ± 1.98

KK 008, *L. paracasei*; KK 033, *L. paracasei*; DG 059, *P. pentosaceus*; DG 068, *L. buchneri*; KK 160, *L. paracasei*

Antibiotic susceptibility of five selected LAB strains was determined both by disc diffusion technique as well as by microdilution method. On the basis of the disc diffusion assay results, it can be concluded that selected LAB strains showed high or medium sensitivity to eight out of nine tested antibiotics in tested concentrations. All selected LAB isolates were sensitive to erythromycin, clindamycin, tetracycline and chloramphenicol in tested concentrations. Differences in the results of LAB isolates susceptibility to tested antibiotics were observed for ampicillin, gentamycin and streptomycin. *L. paracasei* isolates no. KK 008 and KK 033 were susceptible to these three antibiotics. *P. pentosaceus* DG 059 exhibited susceptibility towards ampicillin and intermediate susceptibility for the gentamycin and streptomycin in tested discs concentrations. *L. buchneri* DG 068 was susceptible to gentamycin and intermediate susceptible to ampicillin and streptomycin in tested concentrations. Finally, *L. paracasei* KK 160 demonstrated susceptibility towards ampicillin and streptomycin whereas towards gentamycin these bacteria were intermediate susceptible. Kanamycin (30 mcg) was the only antibiotic for which an intermediate susceptibility of the five LAB isolates was observed. All of the selected isolates were resistant to vancomycin (30 mcg). The results of antibiotics MIC values confirmed previously obtained results. According to the results, all five tested LAB strains were susceptible to ampicillin, gentamicin, kanamycin, streptomycin, erythromycin, clindamycin, tetracycline and chloramphenicol. The MIC values of all five selected LAB strains were lower than the EFSA breakpoints (Table 4). In the EFSA guidelines, cut-off values for *L. buchneri* are not discussed individually, therefore, the respective MIC values were interpreted using breakpoints given for the obligate fermentative *Lactobacillus* bacteria (European Food Safety Authority (EFSA) 2012).

Selected LAB strains showed proteolytic activity, while only two of them: *L. paracasei* KK 008 and KK 160 demonstrated amylolytic activity. None of the five selected LAB exhibited lipolytic properties. Results concerning the production of dominant organic acid, cells hydrophobicity and survival of selected LAB in the GIT conditions are presented in Table 5. Lactic acid production by the studied bacteria ranged from 15.15 to 31.69 mg/mL, depending on the strain. The possibility of adhesion to the epithelium was estimated based on the assessment of the hydrophobicity of cells from 22.44 to 41.61%. Selected isolates were characterized by good tolerance of low medium pH: 2.0–3.0 and bile salts concentration: 0.25–1.0%.

The results of in vitro LAB survival in the GIT conditions demonstrating tolerance of acidic pH and ox bile, are presented on Fig. 3. Growth rate in unfavorable conditions was observed between 3 and 24th h. All the finally selected LAB showed the capacity to survive the tested values of acidic pH: however, their growth was strongly reduced. In case of four out of five LAB, their viability degree was better in pH 3 compared to pH 2. Isolates no. KK 008, KK 033 and KK 160 showed good tolerance of all tested ox bile concentrations, while isolate no. DG 059 only in concentration 0.25%. *L. buchneri* DG 068 was the only vulnerable isolate to the tested concentrations of bile salts.

### Preparation and stability of feed additive

#### Preparation of feed additive using selected LAB isolates

**Fig. 4:**
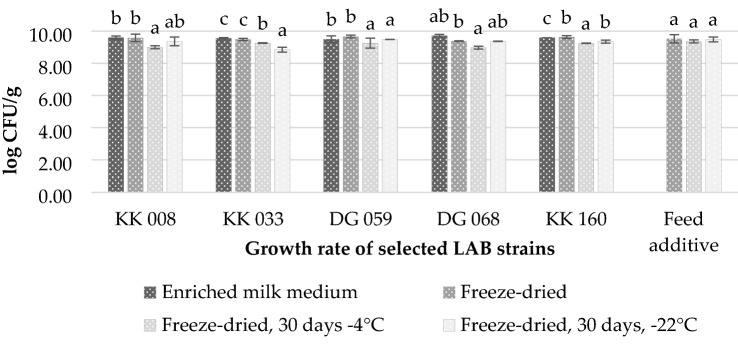
Growth rate of selected LAB strains at each stage of feed additive formulation: KK 008 *L. paracasei*; KK 033, *L. paracasei*; DG 059, *P. pentosaceus*; DG 068, *L. buchneri*; KK 160, *L. paracasei*; feed additive, freeze-dried bacterial cultures of KK 008, KK 033, DG 059, DG 068 and KK 160, ground and mixed thoroughly in equal proportions. Data were analysed by Tukey’s test at *p* < 0.05 (a, b means marked with different letters in bars differ significantly)

Selected LAB isolates, with potentially probiotic properties whose biological activity could complement each other were used for the composition of feed additive prototype for pigs (Fig. 4). Selected LAB strains were cultivated on a medium with milk enriched with trehalose and maltodextrin and freeze-dried. The largest decrease in the population size was observed for *L. buchneri* DG 068 samples (0.34 log CFU/mL). In the remaining samples, the difference was small and ranged from 0.08 to 0.23 log units. The exception was the population of *P. pentosaceus* DG 059, which increased after the fixation process (9.5–9.66 log CFU/mL). The tested LAB showed a high survival rate during freeze-drying, which confirms a proper selection of the medium.

#### The stability of freeze-dried LAB cultures and additive during storage

An important parameter determining the technological usefulness of microorganisms is the stability of the population during storage. The freeze-dried culture samples of selected five LAB, as well as the prepared feed additive, were stored at 4 °C and – 20 °C for 30 days. Next, the number of LAB population was determined. The number of LAB in the mixed feed additive remained stable during storage in different conditions, while in LAB samples statistically significant differences were observed. In all the samples stored at 4 °C the number of LAB was significantly lower than in freeze-dried samples. Among samples stored at − 20 °C, the decrease of population was observed only for *L. paracasei* KK 033 and KK 160.

### Survival of LAB present in feed additive in the simulated digestive system

**Fig. 5:**
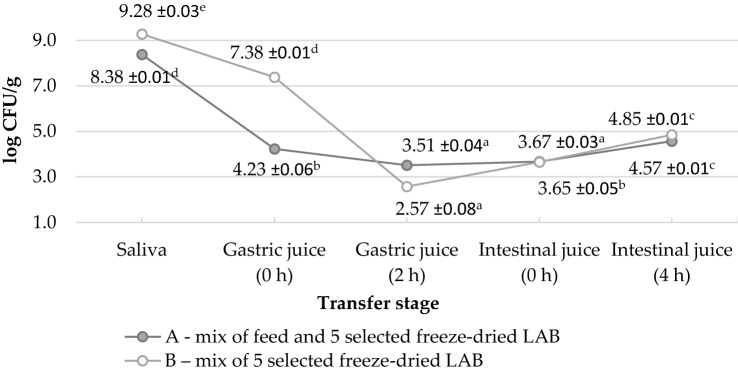
Number of LAB during the passage in the in vitro digestive tract of pigs. Five selected and freeze-dried LAB: KK 008, *L. paracasei*; KK 033, *L. paracasei*; DG 059, *P. pentosaceus*; DG 068, *L. buchneri*; KK 160, *L. paracasei.* Data were analysed by Tukey’s test at *p* < 0.05 (a–e means marked with different letters differ significantly)

Composed prototype of feed additive for pigs has been passaged through the in vitro digestive tract. The experimental conditions were similar to those prevailing in the GIT of monogastric animals. Samples of freeze-dried bacterial cultures of KK 008, KK 033, DG 059, DG 068 and KK 160, ground and mixed thoroughly in equal proportions with commercial feed addition (A) and without the feed (B) were transferred through solutions simulating: saliva, gastric juice and intestinal juice (Fig. 5). The initial number of bacteria tested in samples differed by one logarithmic unit, due to the fact that sample A (8.38 ± 0.01 log CFU) contained addiction of feed, while sample B (9.28 ± 0.03 log CFU) was freeze-dried LAB. After a few minutes of simulated solutions of saliva and gastric juice impact, the number of LAB decreased by 4 and 3 log units (sample A and B, respectively). After two hours of incubation in solution simulating gastric juice, a further decrease in cell population was observed in both samples, by 1 and 3 log units (sample A and B, respectively). After changing the environment to mixture simulating intestinal juice, the number of bacteria equaled in both samples. An increase in LAB population in sample B was observed, while in the sample A this parameter remained at a comparable level. After three more hours of incubation in solution simulating intestinal juice, a significant increase in the number of bacteria was observed in both samples.

## Discussion

LAB are the largest and best characterized group of probiotics. Their beneficial effect is associated with the ability to inhibit pathogens growth, influence the balance of gastrointestinal microbiota and support the immune system. Despite a lot of research concerning probiotics, there is a need to search for new strains as probiotic properties are strain-specific (Casarotti et al. 2017) and many authors suggest linking the origin of the strain and its intended use (Chiang et al. 2015; Bautista-Gallego et al. 2017; Dowarah et al. 2017). In the presented work, evaluation of functional properties of LAB strains isolated from pig feces obtained from suckling and weaned pigs has been carried out according to guideline recommended by FAO/WHO (2002) together with a construction of feed additive prototype. Out of the initial number of 376 isolates, five strains which demonstrated the best properties (including broad range of antimicrobial activity, high production values of organic acids, good survival under GI conditions as well as cells hydrophobicity) have been chosen. The research covered the sequence of experiments with antimicrobial activity as a first criterion. Out of 376 isolates, 87 demonstrated no antibacterial properties. Similar results were reported by other authors who observed up to half of isolates without activity during their screening tests (Guo et al. 2010; Lo Verso et al. 2018). Taking into account that only a part of isolates inhibited the growth of pathogenic bacteria, this step enabled selection of the most promising LAB isolate, tested next for their antibiotic sensitivity. According to EFSA, studies on antibiotic resistance profiles and transmission mechanisms are necessary to give QPS status to probiotic strains intended for industrial use (European Food Safety Authority Panel on Biological Hazards (BIOHAZ) 2013). In the presented study high susceptibility of almost all the tested LAB isolates to chloramphenicol, clindamycin, erythromycin and tetracycline was detected. Over 90% of isolates were highly or intermediate sensitive to streptomycin and ampicillin, more than 70% were sensitive to gentamicin, while high and moderate susceptibility to kanamycin was demonstrated only by 16.44% and 38.36%, respectively. Only two isolates showed sensitivity to vancomycin. These results are consistent with literature data. Moreover, re-evaluation of five finally selected LAB isolates using broth microdilution assay confirmed their susceptibility towards ampicillin, gentamicin, kanamycin, streptomycin, erythromycin, clindamycin, tetracycline and chloramphenicol on the basis of EFSA FEEDAP Panel guidance (European Food Safety Authority (EFSA) 2012). In the present study for microdilution antibiotic testing, LSM was used to avoid the antibiotic-inhibitory activity by the medium components, observed for MRS (Huys et al. 2002; Klare et al. 2005). LAB to grow require carbohydrates, vitamins, minerals and amino acids, so the usage of Mueller Hinton or Iso-Sensitest media is inadequate. Therefore, for nonenterococcal LAB, the combination of Iso-Sensitest and MRS media was obtained by Klare et al. (2005) and further on become a reference medium for microdilution antibiotics tests (International Organization for Standardization (ISO) 2010). In the research of de Souza et al. (2019) all the tested LAB strains were susceptible to ampicillin, erythromycin, clindamycin, tetracycline and chloramphenicol, while all LAB strains were resistant to vancomycin and 85% of strains were resistant to kanamycin. Similarly, Birri et al. (2012) reported that all the tested *L. casei* strains were resistant to vancomycin and susceptible to erythromycin, chloramphenicol and tetracycline. In the study of Jeronymo-Ceneviva et al. (2014) 100% of LAB strains isolated from cheese showed resistance to vancomycin. In turn, Casarotti et al. (2017) reported that all the *L. delbrueckii* subsp. *bulgaricus* strains were susceptible to vancomycin and gentamicin, whereas other strains, belonging to *L. rhamnosus*, *L. casei* and *L. fermentum* species, were resistant. The resistance of LAB to vancomycin, according to some authors, will not be transferable to pathogenic bacteria as it is an intrinsic feature, codified by chromosomic genes (Divya et al. 2012) and the replacement of terminal d-alanine by d-lactate or d-serine in the muramyl-pentapeptide prevents vancomycin binding (Gueimonde et al. 2013). As the antibiotic sensitivity was an important criterion, it allowed to choose 41 isolates for further characterization including organic acids production, enzymatic activity, hydrophobicity as well as tolerance of acid and bile salts.

Determination of the content of organic acids, such as lactic and acetic acids, which are the main metabolites of LAB, was, on the one hand, confirmation that the tested isolates belong to this group of microorganisms, and, on the other hand was one of the selection factors. Moreover, propionic acid content was also determined, having considered that some heterofermentative or optionally heterofermentative LAB may produce it (Ripamonti et al. 2011). High level of lactic acid production is a desirable feature for commercial-use strains selection, as their functional properties, e.g. antimicrobial activity, are dependent, among others, on the ability of bacteria to acidify their habitat. Although differentiated mechanisms may play a role in the inhibition of pathogens, such as competitive exclusion and production of antimicrobial substances including bacteriocins or hydrogen peroxide, organic acids are important metabolites involved in the antagonistic activity (Niku-Paavola et al. 1999; Arena et al. 2016; Sirichokchatchawan et al. 2018).

The functional characteristics of LAB isolates included the enzymatic properties as a particularly desirable feature in supplements intended for animals, supporting digest feed (Lee et al. 2001). Many authors emphasize the desirability of testing microorganisms in terms of determining enzymatic properties during the selection of strains for animal feed supplementation. In the presented study, rather weak enzymatic activity of LAB isolates has been stated. None of the tested bacteria showed lipolytic activity and some of them showed only weak proteolytic and amylolytic properties. Similar amylolytic activity results were obtained by Guo et al. (2010), where only 3 LAB isolates out of 150 tested showed high capacity of starch digestion. Literature data indicate that the enzymatic properties of LAB are very diverse. Similar results were obtained by Kim et al. (2007) who tested 252 LAB isolates derived from faeces and intestines of piglets in terms of their ability to produce proteases, amylases, lipases and phytases. 81% of the strains tested had proteolytic properties, 22% of isolates derived from feces and 23% obtained from intestines had amylolytic properties. However, the authors obtained different results for lipolytic properties, which they found in 15% of faeces isolates and 17% obtained from intestines. Differences in the occurrence of certain enzymatic properties among LAB may result, among others, from their origin. For example, authors describe strains that have strong amylolytic properties that come from fermented starchy foods (Sanni et al. 2002). Another problem affecting the biochemical properties of bacteria may be the degree of binding of enzymes to the cell. (Lee et al. 2001) during selection of potentially probiotic bacteria found that the amylolytic activity of *L. acidophilus* L23 was closely related to the presence of cells, which indicates that the enzyme was associated with the cell wall. In turn, another strain, *L. fermentum* L9, produced extracellular amylolytic enzymes.

Among the most important parameters determining the usefulness of LAB as potential probiotics, the hydrophobicity as well as tolerance of low pH and bile salts should be taken into account. Cell surface hydrophobicity affects the overall adhesion capacity and can facilitate the contact between bacterial and host epithelial cells (Kos et al. 2003; Sánchez-Ortiz et al. 2015). According to literature data, high hydrophobicity indicates that bacteria can bind better to the intestinal mucosa (Todorov et al. 2011; de Souza et al. 2019), therefore, the adhesion to hydrocarbons is used as a biochemical marker allowing to evaluate this feature (Gandomi et al. 2019). In the presented work various hydrophobicity among tested LAB isolates was observed with the dominance of isolates demonstrating low degree of adhesion to toluene and only 7 out of 41 isolates showing high hydrophobicity. These results are consistent with data reported by other authors. (Angmo et al. 2016) have stated extremely variable results of tests provided for 25 isolates, where only 3 demonstrated high hydrophobicity. Generally low degree of hydrophobicity with 5 out of 19 strains obtained from Mozzarella cheese showed values around 60% observed (de Souza et al. 2019). In turn, (Jeronymo-Ceneviva et al. 2014) noticed high hydrophobicity value for all four tested LAB strains isolated from water-buffalo Mozzarella cheese. LAB isolates tolerance to ox bile and acidic pH was studied to predict bacterial survival after oral administration. Extreme conditions of the medium set at the 2 and 3 pH as well as 1.00%, 0.50% and 0.25% ox bile concentration were used to reflect stomach prevailing conditions (Lin et al. 2007; Guo et al. 2010; Pérez-Sánchez et al. 2011b). Guo et al.(2010) reported that, piglets originating, LAB isolates losses the viability due to the high acidity of the medium as well as tested bile concentrations. This phenomenon was also observed in our studies in case of a few isolates. However, in this paper, the majority of the tested LAB isolates revealed tolerance to acidic pH and tested ox bile concentrations for 3 h, no significant decrease of viable counts was observed, even bacterial growth was detected after 24 h. All tested by García-Hernández et al. LAB isolates from broiler chicken showed resistance to acidic environment (pH 2.5), although bacterial growth was depressed. Bile salts concentration of 0.3% occurred in obtaining very different results of LAB survival after 3 h of exposure and bacterial final growth after 12 h of incubation. The strain LB-31 showed the highest value of 82.13% for initial survival, but the lowest relative final growth. Obtained by several studies results suggest that the acid tolerance of LAB isolates was strain-specific (Mishra and Prasad 2005; Guo et al. 2010).

Freezing with cryoprotectants at low temperatures is a common method applied to preserve the viability and properties of LAB during long-term storage. One of the most effective cryoprotectant, which is widely used in frozen concentrates, is glycerol. Although glycerol prevents bacteria from freezing damage, their viability after storage decreases. The mechanisms of bacterial freezing damage are not completely understood. Mainly low freezing temperature, ice crystal formation as well as glass transitions are causing cellular damage due to occurring cell stresses including cold, osmotic, mechanical as well as oxidant stress. During freezing, damages of membrane lipids, cell proteins and DNA is observed, leading to cell functionality losses and finally loss of viability (Fonseca et al. 2006, 2015b; Smirnova et al. 2019). The bacterial viability as well as their biological activity after freezing and frozen storage depends on: the strain, culture conditions, cryoprotectants and their formulations, freezing conditions and storage time (Fonseca et al. 2001, 2006; Smith and Ryan 2012). In the presented paper, long-term storage of tested LAB isolates was performed according to the protocol with 80% glycerol, the freezing formulation ratio was 1:1 cryoprotectant solution with freshly cultivated 24 h LAB cultures. After 18 months of frozen storage, in most cases LAB isolates maintained antagonistic effect against indicator bacteria, compared to the results obtained in previous antimicrobial tests. However, both reduction and disappearance of LAB biological activity were observed. This was in accordance with results previously reported by Fonseca et al. (2006), where the viability as well as acidification properties decreased after freezing storage.

The selected five LAB isolates were identified at both the genus and species level by MALDI-TOF MS and 16S rRNA gene sequencing. Chosen LAB isolates were identified as *Lentilactobacillus buchneri* (DG 068), *Pediococcus pentosaceus* (DG 059) as well as *Lacticaseibacillus paracasei* (KK 008, KK 033, KK 160). *Lacticaseibacillus* group (LCG) includes phenotypically as well as genotypically closely related species as *L. casei*, *L. paracasei* and *L. rhamnosus*, therefore, identification and differentiation of these probiotic strains using 16S rRNA gene sequencing is burdened by obtaining ambiguous results, due to inability to discriminate between species of very high sequence homology. Therefore, the Authors also used MALDI-TOF mass spectrometry as tool for identification of isolated LAB (Bizzini and Greub 2010; Dec et al. 2016). As the literature data states, MALDI-TOF MS using proteomics based identification, is able to discriminate between bacterial strains at both the genus and species levels and subspecies. It is a simple, fast, accurate and affordable method of identification (not including initial equipment costs) (Spinali et al. 2015; Tran et al. 2015; Huang et al. 2018). Using, 16S rRNA gene sequencing, in presented studies three selected LAB isolates were identified as *L. paracasei* species. The results were inconclusive on the subspecies level identification, indicating homology of isolated strains to *L. paracasei subsp. tolerans*, *L. paracasei subsp. paracasei* or just to *L. paracasei*. Identification based on the MALDI-TOF MS gave clear results, identifying these three LAB strains, on species level, as *L. paracasei.* To increase identification rates, as well as for obtaining identification on subspecies level, through matching the expansion of the BioTyper database is indispensable. (Dušková et al. 2012), also obtained ambiguous results, as two out of 148 *Lactobacillus* strains characterized by PCR and MALDI-TOF MS were, as the Authors stated, incorrectly assigned to *L. paracasei* and *L. zeae*. The Authors indicated that obtained results are related to the presence of species limitated entries in the BioTyper database as well as unclear *Lactobacillus* spp. taxonomic description.(Dec et al. 2014) also observed that the MALDI-TOF MS identification of the *Lactobacillus* species resulted with more than one assignment for 11.5% of tested strains, mostly in case of *L. johnsonii*. Therefore, it is clear that both 16S rRNA gene sequencing as well as MALDI-TOF MS are not infallible as they failed to differentiate between *L. casei* and *L. paracasei*. *Lacticaseibacillus* sp. can be identified with the greatest accuracy using approach which combines several genotypic methods (Huang et al. 2018).

Finally, the prototype of feed additive was prepared based on the selected LAB strains and survival tests during freeze-drying, storage as well as during the passage through the simulated GIT were carried out. Freeze-drying is commonly used as a preservation method; however, the stress factors can cause undesirable loss of viability of probiotics, therefore, different cryoprotectants are applied to increase their survival rate during the procedure (Siaterlis et al. 2009; Tomás et al. 2009). The mostly studied protectants include skim milk, whey proteins, varied sugars, or other bio-polymers (Meng et al. 2008; Han et al. 2018). In the presented study, the milk medium with trehalose and maltodextrin was used for LAB culture subjected to freeze-drying, which allowed to obtain good survival of bacteria during lyophilization and storage; however, both processes affects the cells viability. This is consistent with literature data indicating that some compounds such as trehalose, used in the presented study, as well as maltodextrin, glucose or sucrose may affect the viability during freeze-drying and storage (Strasser et al. 2009; Ren et al. 2019). Also, the effect of temperature during storage was underlined in literature data, which has been stated in the presented work. The stability of freeze-dried samples decreases during storage, and higher survival rates are recorded at lower storage temperatures. For example, (Ren et al. 2019) observed better viability of two *Lactobacillus* strain (*L. salivarius* and *L. agilis*) in the temperature of 4 °C in comparison with room temperature. Also, (Deeseenthum et al. 2007) reported that probiotic *Bacillus* spp. strains were more stable at the temperature of 4 °C than in room temperature. Moreover, it is worth underlining that the effects of storage may be highly variable and strain dependent (Turuvekere Sadguruprasad and Basavaraj 2018). Similar results were noticed in the presented study.

The feed additive prototype composed of five selected strains in two forms, as freeze-dried cultures and freeze-dried cultures mixed with feed, has been passaged in the in vitro digestive tract, where similar tendency was observed for both of them. The viability of LAB decreased in simulated solution of gastric juice and increased in simulated mixture of intestinal juice, which complies with other studies in literature. (Guerra et al. 2007) tested in vitro the survival of four strains including *Pediococcus acidilactici*, *Enterococcus faecium*, *L. casei* and *L. lactis* in the GIT and observed a decrease in the viable cells population during the passage through the stomach, while after 180 min of passage including the intestinal part the number of living cells was at a level of 10^6^ CFU/mL. Similarly, Simões da Silva et al. (2020) observed significant decrease in the viability of different LAB strains in fermented products made of buffalo and cow milk after the gastric phase. The survival rate of bacteria may depend on the form in which they are administered, taking into account both the form of preservation and the type of matrix in which they are applied (Klingberg and Budde 2006). It has been found that the survival of LAB in the stomach can increase in the presence of food products that affect the pH value and can protect cells from the effects of pepsin and acid in the stomach (Bergamini et al. 2005). Ranadheera et al. (2012) have stated higher survival rate of *L. acidophilus* in ice cream and goat milk yogurt with 10% fat, when they were transferred through GIT. Also, Simões da Silva et al. (2020) reported that viability of LAB strains during the gastrointestinal simulation test remained more viable in dairy products made with buffalo milk compared to cow milk. In this study, however, a significant effect of the matrix was not observed, although in some sections of the simulated GIT, greater stability of the LAB population in the presence of feed could be observed.

## Conclusions

LAB populations are specific as well as unique for different animals, therefore, bacterial isolation from suckling and weaned pigs was carried out to compose a multi-species probiotic feed additive prototype for swine. Functional properties of the obtained isolates varied significantly, therefore, selection of LAB isolates characterized by complementary features took place. Finally chosen 5 LAB isolates presenting satisfactory probiotic properties may be exploited as host specific probiotics for swine. The biggest advantage lies in the selection of functional microorganisms to maintain the relationship of bacterial origin with the group of monogastric animals for which the additive would be dedicated. All the research was conducted in vitro, therefore, further in vivo trials should proceed to assess the effect of the feed additive prototype on the performance and health of pigs. Preliminary studies for multistrain feed additive as well as animal feeding tests should be designed and performed before the field feeding use.

## Supplementary Information

Below is the link to the electronic supplementary material.

Supplementary file1 (DOCX 17 kb)
