# Supplementary material for: Isolation and probiotic potential of lactic acid bacteria from swine feces for feed additive composition
Source: Arch Microbiol. 2021 Dec 23;204(1):61. doi: 10.1007/s00203-021-02700-0 (PMC8702511; doi:10.1007/s00203-021-02700-0)
Supplement: Supplementary file 1 — Supplementary file1 (DOCX 17 kb) [file 203_2021_2700_MOESM1_ESM.docx]

**Supplementary material**

**Isolation and probiotic potential of Lactic Acid Bacteria from swine feces for feed additive composition**

**Katarzyna Marchwińska** (ORCID: 0000-0001-9546-5334)**,**

**Daniela Gwiazdowska** (ORCID: 0000-0002-0972-6225)

*Department of Natural Science and Quality Assurance, Institute of Quality Science,*

*Poznań University of Economics and Business, Poland*

Corresponding author: katarzyna.marchwińska@ue.poznan.pl

*Table S1. Antibacterial activity of selected LAB isolates derived from the piglets feces.*

| **Indicator  bacteria** | **Isolates with specific antibacterial properties** | | | | | | | |
| --- | --- | --- | --- | --- | --- | --- | --- | --- |
|  | **Strong** | | **Moderate** | | **Weak** | | **Lack of impact** | |
|  | **>20.1 mm ^1^** | | **20.0-15.1 mm ^1^** | | **<15.1 mm ^1^** | |  |  |
|  | n | % | n | % | n | % | n | % |
| *C. perfringens* | 6 | 1.60 | 29 | 7.71 | 56 | 14.89 | 103 | 75.80 |
| *L. monocytogenes* | 0 | 0.00 | 7 | 1.86 | 45 | 11.97 | 119 | 86.17 |
| *S. aureus* | 17 | 4.52 | 42 | 11.17 | 72 | 19.15 | 78 | 65.16 |
| *E. aerogenes* | 6 | 1.60 | 36 | 9.57 | 71 | 18.88 | 101 | 69.95 |
| *E. coli* | 2 | 0.53 | 36 | 9.57 | 47 | 12.50 | 97 | 77.39 |
| *P. vulgaris* | 0 | 0.00 | 1 | 0.27 | 51 | 13.56 | 117 | 86.17 |
| *S.* Enteritidis | 0 | 0.00 | 1 | 0.27 | 54 | 14.36 | 117 | 85.37 |
| *S.* Typhimurium | 2 | 0.53 | 46 | 12.23 | 44 | 11.70 | 103 | 75.53 |
| *S. marcescens* | 8 | 2.13 | 36 | 9.57 | 79 | 21.01 | 92 | 67.29 |
| *Y. enterocolitica* | 0 | 0.00 | 1 | 0.27 | 31 | 8.24 | 122 | 91.49 |

^1^ inhibition zone diameter; n – number of LAB isolates, % - percentage of LAB isolates.

*Table S2. The sensitivity of the selected 71 LAB isolates derived from the piglets feces to antibiotics.*

| **Antibiotic** ^1^ | (mcg) | **Susceptibility** | | **Intermediate  susceptibility** | | **Resistance** | |
| --- | --- | --- | --- | --- | --- | --- | --- |
|  |  | n | % | n | % | n | % |
| **ampicillin** | **10** | 52 | 73.24 | 18 | 25.35 | 1 | 1.41 |
| **vancomycin** | **30** | 2 | 2.82 | 0 | 0.00 | 69 | 97.18 |
| **gentamicin** | **10** | 39 | 54.93 | 12 | 16.90 | 20 | 28.17 |
| **kanamycin** | **30** | 12 | 16.44 | 28 | 38.36 | 33 | 45.21 |
| **streptomycin** | **300** | 49 | 69.01 | 18 | 25.35 | 4 | 5.63 |
| **erythromycin** | **15** | 70 | 98.59 | 1 | 1.41 | 0 | 0.00 |
| **clindamycin** | **2** | 71 | 100.00 | 0 | 0.00 | 0 | 0.00 |
| **tetracycline** | **30** | 65 | 91.55 | 5 | 7.04 | 1 | 1.41 |
| **chloramphenicol** | **30** | 69 | 97.18 | 1 | 1.41 | 1 | 1.41 |

^1^ concentration; n – number of LAB isolates, % - percentage of LAB isolates.
